# Prevalence of childhood obstructive sleep apnea syndrome and its role in daytime sleepiness

**DOI:** 10.1371/journal.pone.0204409

**Published:** 2018-10-03

**Authors:** Eriko Tsukada, Shingo Kitamura, Minori Enomoto, Aiko Moriwaki, Yoko Kamio, Takashi Asada, Tetsuaki Arai, Kazuo Mishima

**Affiliations:** 1 Department of Psychophysiology, National Institute of Mental Health, National Center of Neurology and Psychiatry, Kodaira-city, Tokyo, Japan; 2 Department of Psychiatry, Graduate School of Comprehensive Human Sciences, University of Tsukuba, Tsukuba-city, Ibaraki, Japan; 3 Department of Psychiatry, University of Tsukuba Hospital, Tsukuba-city, Ibaraki, Japan; 4 Support Room for Students with Disabilities, Tokyo Gakugei University, Koganei-city, Tokyo, Japan; 5 Department of Child and Adolescent Mental Health, National Institute of Mental Health, National Center of Neurology and Psychiatry, Kodaira-city, Tokyo, Japan; 6 Department of Neuropsychiatry, University of Tokyo Medical and Dental University, Bunkyo-ku, Tokyo, Japan; 7 Department of Psychiatry, Faculty of Medicine, University of Tsukuba, Tsukuba-city, Ibaraki, Japan; 8 Department of Neuropsychiatry, Akita University Graduate School of Medicine, Akita-city, Akita, Japan; Chiba Daigaku, JAPAN

## Abstract

**Objectives:**

To investigate childhood obstructive sleep apnea syndrome (OSAS) and its role in daytime sleepiness among school-age children.

**Methods:**

A questionnaire survey was conducted with 25,211 children aged 6–15 (mean, 10.39) years attending 148 elementary and 71 middle schools in 10 prefectures across Japan and their parents. Questions concerned 4 sleep habit items (bedtime, sleep onset latency, wake time after sleep onset, wake-up time) and 4 sleep disorder items (loud snoring, snorts/gasps, breathing pauses, seems very sleepy in the daytime). Total sleep time (TST) was calculated with sleep habits. Severe possible OSAS (p-OSAS) was defined as having loud snoring, snorts and gasps, or breathing pauses “frequently” (≥ 5 times per week), and mild p-OSAS was rated as having any of these “sometimes” (2–4 times per week). Severe daytime sleepiness was defined as seeming very sleepy “frequently” and mild daytime sleepiness as seeming very sleepy “sometimes”.

**Results:**

Mean prevalence of mild to severe p-OSAS and severe p-OSAS in children across all grade levels was 9.5% and 1.6%, respectively. p-OSAS was particularly prevalent in children at lower elementary levels, decreasing with advancing grade levels. Prevalence of mild and severe daytime sleepiness was 6.1% and 0.9%, respectively, among all children (7.0%). Prevalence of daytime sleepiness increased with advancing grade levels, particularly in middle-school level. Average TST was 8.4 ± 2.2 h in both elementary and middle-school levels, and decreased as grades advanced, particularly in middle-school levels. Multivariate logistic regression analysis showed that middle-school level, TST < 8 h, and p-OSAS were independent factors for daytime sleepiness. Strong correlations were found between severe daytime sleepiness and severe p-OSAS or TST < 6 h, and between daytime sleepiness and loud snoring or breathing pauses.

**Conclusion:**

p-OSAS may be an independent factor influencing daytime sleepiness in school-age children. Loud snoring and breathing pauses could be clinical markers for children with severe daytime sleepiness.

## Introduction

Sleep-related problems are frequently encountered during childhood developmental stages. Approximately one-fourth of children have some form of sleep problems [1], with daytime sleepiness being extremely prevalent. The intensity and frequency of sleepiness vary among studies, depending on the definitions used, but it is known that 30–40% of school-age children are frequently aware of feeling very sleepy in the daytime [2, 3], which greatly exceeds the proportion among adults (5–15%) [4–6].

Insufficient sleep is the leading cause of sleepiness in children. Annual surveys on sleep habits show that children’s sleep duration continues to shorten in the United States, Europe, Asia, and Oceania [7–13]. In school-age children, sleep duration shortens as they move through school grade levels [14]. Given that wakeup times do not change across grade levels, this shortened sleep duration is presumably because bedtime becomes progressively later, leading to insufficient sleep, daytime sleepiness, classroom napping, and even social dysfunction and poor quality of life [14].

The high prevalence of daytime sleepiness in children may also be caused by sleep disorders such as sleep apnea syndrome, restless legs syndrome, periodic limb movement disorder, and sleepwalking [15, 16]. Among these disorders, obstructive sleep apnea syndrome (OSAS) is of particular clinical relevance due to its high incidence rate [17]. The prevalence of OSAS and other sleep-related breathing disorders among children is estimated to be 1–5.8% by definitive diagnostic surveys using objective indicators such as polysomnography or pulse oximetry [18–20] and 4–11% based on questionnaire surveys of parents [21, 22]. OSAS causes various mental and physical problems in children, as it does in adults. Childhood OSAS is a strong risk factor for childhood developmental problems. It is strongly associated with neurobehavioral problems, such as sleepiness, impaired attention, hyperactivity, learning disorder, memory impairment, poor academic performance, and depression [23–25], and is a risk factor for physical problems, such as growth impairment, developmental delay [26], cardiovascular comorbidities [27], metabolic disorders and inflammation [28, 29].

Although OSAS is known to cause excessive daytime sleepiness in adults, the degree of its involvement in daytime sleepiness among children is controversial [30]. The frequency of daytime sleepiness in children with OSAS varies widely, ranging from around 10% to 50%, depending on the survey methods [31–33], and no consensus has been reached on whether OSAS is a major cause of sleepiness among children. To address this, we conducted a large-scale epidemiological study of mental health and sleep state to reveal the incidence of daytime sleepiness and OSAS among school-age children and the extent of OSAS involvement in sleepiness, independent of sleep duration.

## Materials and methods

### Subjects

The subjects of this study to reveal sleep-related problems in school-age children were 87,578 regular students attending 148 elementary schools and 71 middle schools in 10 prefectures across Japan (Hokkaido, Akita, Saitama, Nagano, Toyama, Ishikawa, Fukui, Shiga, Tokushima, and Saga). In Japan, compulsory education is for 6 years in elementary school (grades 1–6) and 3 years in middle school (grades 7–9).

### Survey method

Schools distributed two questionnaire forms to parents between December 2009 and February 2010. Parents who consented to participate returned the questionnaire by postal mail. Questionnaire forms returned by 30 April 2010 were used for data analysis. This study was approved by the Ethics Committee of the National Center of Neurology and Psychiatry and was conducted in compliance with Japan’s Ethical Guidelines for Epidemiological Research.

### Questionnaire items

Parents completed questionnaire items about their children’s sleep habits and sleep-related problems during the past 1 month.

Sleep habits were evaluated using a questionnaire form with 9 questions developed based on the Brief Screening Questionnaire for Infant Sleep Problems [34]. The 9 items gathered data on the child’s bedtime (BT), bedtime irregularity (on average, 90 min per week), sleep onset latency (SOL, the time needed before falling asleep at night), number and duration of nocturnal awakenings (i.e., wake after sleep onset, WASO), wake time (WT), and the number and duration of daytime naps during the past 1 month. Collected data were used to calculate the following variables: sleep onset time (SOT = BT + SOL), time in bed (TIB = interval from BT to WT), and total night sleep time (TST = TIB–[SOL+WASO]).

Sleep-related problems were evaluated using a previously standardized brief 19-item sleep questionnaire for children [35], developed based on the Children's Sleep Habits Questionnaire [36]: 4 items on bedtime behavior, 12 on behavior occurring during sleep, 5 on problems related to morning awakening, and 2 on daytime sleepiness. In this study, obstructive sleep apnea syndrome and daytime sleepiness were evaluated with the following 4 questions: “Does your child snore loudly?”, “Does your child seem to stop breathing when sleeping?”, “Does your child snort and/or gasp when sleeping?”, and “Does your child seem very sleepy in the daytime” These items were answered using a 3-point Likert scale: 1 rarely (never or 1 time per week), 2 sometimes (2–4 times per week), and 3 frequently (≥ 5 times per week).

### Definitions

Possible OSAS (p-OSAS) was defined as a response of sometimes (2–4 times per week) or frequently (≥ 5 times per week) to at least 1 of the 3 questionnaire items related to OSAS (loud snoring, snorts and gasps, and breathing pauses).

Severity of p-OSAS was evaluated based on the incidence of the related symptoms. Severe p-OSAS was defined as having at least 1 of 3 OSAS-associated symptoms (loud snoring, snorts and gasps, and breathing pauses) “frequently” (≥ 5 times per week), and mild p-OSAS was rated as having any of these “sometimes” (2–4 times per week).

Severity of daytime sleepiness was defined according to answers to the question, “Does your child seem very sleepy in the daytime?” Severe daytime sleepiness was defined as a response of “frequently” and mild daytime sleepiness as a response of “sometimes”, respectively.

### Statistical analysis

Logistic regression analysis was performed with presence of severe or mild daytime sleepiness as the dependent variable and p-OSAS, total sleep time, sex, and grade level as independent variables. Male sex was used as the reference for sex and grade 1 as the reference for grade level. Children were classified by severity of OSA (none, mild, or severe), with none as the reference. TST was divided into < 6 h, 6 to < 7 h, 7 to < 8 h, 8 to < 9 h, 9 to < 10 h, and ≥ 10 h, with 8 to < 9 h used as the reference because the mean TST of children in this study was 8.4 ± 1.1 h.

We performed logistic regression analysis using presence of severe or mild daytime sleepiness as the dependent variable and the 3 OSAS-associated symptoms (loud snoring, snorts and gasps, and breathing pauses), total sleep time, sex, and grade level as independent variables to calculate odds ratios, 95% confidence intervals, and probability. Statistical analysis was performed using SPSS version 21 software (IBM, Tokyo, Japan), with statistical significance set at p < 0.05 for all critical probability levels.

## Results

### Subjects

Parents of 25,779 children responded to the questionnaire survey. Data analysis was conducted on 25,211 children after excluding 568 due to missing or deviated data for sex, grade level, or age.

### Obstructive sleep apnea syndrome

Table 1 shows the prevalence of OSAS-associated symptoms (loud snoring, snorts and gasps, and breathing pauses) and the prevalence of p-OSAS by grade. Among the sleep-related breathing disorders, loud snoring occurred most frequently, as often as 2–4 times a week in 7.2% of students and ≥5 times a week 1.5%. The prevalence of mild/severe and severe p-OSAS was 9.5% and 1.6% across all grade levels. Children in the lower grades of elementary school had the highest rates of sleep-related breathing disorders and p-OSAS, and these rates decreased as grade levels increased. Also, the prevalence of mild and severe p-OSAS was higher in male students than in female students across all grade levels, showing a sex-related difference in the prevalence of p-OSAS.

**Table 1:** Prevalence of OSAS-related symptoms and daytime sleepiness in childhood and adolescence.

		School grade level									TOTAL
		1	2	3	4	5	6	7	8	9	
Loud snoring % (M/F)	Sometimes	8.6	7.8	8.1	7.0	7.6	7.0	6.3	6.0	4.3	7.2
		9.4/7.7	9.1/6.3	9.6/6.5	7.8/6.2	8.3/6.9	7.7/6.3	7.5/5.0	6.6/5.5	5.2/3.2	8.2/6.2
	Frequently	1.7	1.6	1.6	1.5	1.6	1.7	1.6	1.2	0.7	1.5
		2.1/1.3	1.5/1.7	1.5/1.6	2/0.9	2.2/0.9	2.3/1.0	2.2/1.0	1.4/1.0	0.8/0.6	1.8/1.2
	Total	10.3	9.4	9.6	8.5	9.2	8.6	7.8	7.3	5.0	8.7
		11.5/8.9	10.6/8.1	11.1/8.2	9.8/7.1	10.5/7.8	10/7.3	9.7/6.0	8.1/6.5	6.0/3.8	10/7.3
Snorts and gasps % (M/F)	Sometimes	1.5	1.4	1.0	0.6	0.9	0.5	0.6	0.5	0.2	0.9
		2/1.0	1.5/1.2	1.5/0.6	0.9/0.4	1.2/0.6	0.8/0.2	0.7/0.4	0.6/0.3	0.3/0.1	1.2/0.6
	Frequently	0.3	0.1	0.2	0.2	0.2	0.3	0.2	0.1	0.1	0.2
		0.3/0.2	0.2/0.1	0.3/0.1	0.4/0.0	0.2/0.1	0.5/0.1	0.1/0.2	0/0.2	0.1/0.0	0.3/0.1
	Total	1.8	1.5	1.3	0.8	1.1	0.8	0.7	0.6	0.3	1.1
		2.3/1.2	1.7/1.3	1.9/0.7	1.2/0.4	1.4/0.6	1.3/0.3	0.8/0.7	0.6/0.5	0.5/0.1	1.4/0.7
Breathing pauses % (M/F)	Sometimes	1.6	2.0	0.9	1.0	1.2	0.7	0.8	0.5	0.6	1.1
		2.3/0.9	2.3/1.7	1.3/0.5	1.5/0.6	1.6/0.8	0.8/0.6	0.8/0.7	0.7/0.3	0.7/0.5	1.5/0.8
	Frequently	0.2	0.1	0.3	0.2	0.3	0.2	0.2	0.1	0.2	0.2
		0.2/0.2	0.2/0.1	0.3/0.3	0.3/0.1	0.4/0.1	0.4/0.0	0.2/0.2	0/0.3	0.3/0.1	0.3/0.2
	Total	1.8	2.1	1.2	1.3	1.5	0.9	0.9	0.7	0.8	1.3
		2.5/1.1	2.4/1.8	1.7/0.8	1.8/0.7	2.1/1.0	1.2/0.6	1/0.9	0.7/0.6	1/0.6	1.7/0.9
p-OSAS % (M/F)	Mild	9.6	9.2	8.8	7.4	8.2	7.4	6.8	6.3	4.4	7.9
		10.8/8.3	10.6/7.6	10.6/7.0	8.3/6.6	9.1/7.3	8.2/6.5	8.3/5.4	6.9/5.7	5.3/3.3	9.0/6.7
	Severe	1.9	1.7	1.7	1.6	1.8	1.8	1.7	1.3	0.8	1.6
		2.4/1.4	1.6/1.7	1.6/1.9	2.2/1.0	2.4/1.1	2.6/1.0	2.2/1.2	1.4/1.1	0.9/0.7	2.0/1.3
	Total	11.5	10.9	10.5	9.0	10.0	9.2	8.6	7.6	5.2	9.5
		13.2/9.7	12.2/9.3	12.2/8.9	10.4/7.6	11.4/8.4	10.8/7.5	10.5/6.6	8.3/6.8	6.3/4.1	11/7.9
Daytime sleepiness % (M/F)	Mild	4.2	3.4	4.0	4.7	5.0	5.7	10.1	10.8	11.8	6.1
		3.4/5.0	3.6/3.2	4.0/4.1	4.0/5.4	4.2/5.9	5.3/6.1	9.0/11.3	9.8/11.8	10.8/12.8	5.5/6.7
	Severe	0.4	0.2	0.4	0.3	0.7	0.8	1.7	2.3	2.6	0.9
		0.4/0.4	0.2/0.2	0.4/0.4	0.3/0.3	0.6/0.8	1.3/0.3	1.0/2.5	2.3/2.3	2.5/2.6	0.9/0.9
	Total	4.6	3.6	4.4	5.0	5.7	6.5	11.9	13.1	14.3	7.0
		3.8/5.4	3.8/3.4	4.3/4.5	4.3/5.7	4.8/6.7	6.5/6.4	10/13.7	12.1/14.1	13.3/15.4	6.3/7.7
Total sleep time hours (SD)	Total	9.16(0.9)	9.00(0.9)	8.89 (0.8)	8.65 (0.9)	8.43 (1.0)	8.20 (0.9)	7.54 (0.9)	7.32 (1.0)	7.05 (1.1)	8.39 (1.1)
	M/F	9.15(0.9)/ 9.17(0.8)	9.06(0.8)/ 8.94 (1.0)	8.89(0.9)/ 8.89 (0.8)	8.71(0.9)/ 8.59 (0.9)	8.51(0.9)/ 8.35 (1.0)	8.27(1.0)/ 8.14 (0.8)	7.67(0.9)/ 7.41 (0.9)	7.43(0.9)/ 7.2 (1.0)	7.13(1.1)/ 6.96 (1.0)	8.46(1.1)/ 8.32 (1.2)

Sometimes: 2 to 4 days per week; Frequently: ≥ 5 days per week

p-OSAS: possible obstructive sleep apnea syndrome.

Total sleep time is expressed as mean (SD).

### Prevalence of daytime sleepiness

Mild and severe daytime sleepiness was reported in 6.1% and 0.9% of school-age children, respectively, and in 7.0% of all children (Table 1). The prevalence of daytime sleepiness increased with advancing grade levels and was especially notable in middle-school levels. Daytime sleepiness was more prevalent across all grade levels in girls than boys (6.3% and 7.7%, respectively).

### Total sleep time

TST was 8.4 ± 2.2 h in all children (Table 1). TST decreased with advancing grade levels and was particularly apparent in middle-school levels.

### Risk factors for daytime sleepiness

Multivariate logistic regression analysis revealed the independent factors associated with both mild and severe daytime sleepiness were middle-school level, TST < 8 h, and presence of p-OSAS (Table 2). Specifically, middle-school level, severe p-OSAS, and TST < 6 h were more strongly associated with severe daytime sleepiness than with mild daytime sleepiness.

**Table 2:** Factors associated with daytime sleepiness in childhood and adolescence.

			Mild sleepiness		Severe sleepiness	
			Odds ratio	(95% CI)	Odds ratio	(95% CI)
Sex	Male		−	−	−	−
	Female		1.23 ^#^	(1.10–1.37)	1.04	(0.79–1.37)
Grade level	Elementary school	1	−		−	−
		2	0.77	(0.60–1.00)	0.55	(0.22–1.36)
		3	0.89	(0.69–1.15)	1.08	(0.50–2.33)
		4	1.01	(0.79–1.30)	0.77	(0.33–1.77)
		5	0.98	(0.77–1.26)	1.54	(0.75–3.13)
		6	1.04	(0.81–1.34)	1.38	(0.66–2.89)
	Junior high school	7	1.44 *	(1.12–1.86)	2.07 ^$^	(1.02–4.21)
		8	1.48 *	(1.14–1.91)	2.21 ^$^	(1.09–4.49)
		9	1.47 *	(1.12–1.92)	2.19 ^$^	(1.06–4.52)
p-OSAS	Absent		−	−	−	−
	Mild		2.32 ^#^	(1.98–2.72)	2.43 ^#^	(1.62–3.65)
	Severe		2.25 ^#^	(1.62–3.13)	9.14 ^#^	(5.65–14.77)
Total sleep time (h)	< 6		2.23 ^#^	(1.69–2.94)	11.45 ^#^	(6.92–18.96)
	6–7		2.54 ^#^	(2.07–3.10)	5.08 ^#^	(3.11–8.32)
	7–8		1.44 ^#^	(1.23–1.69)	2.13 ^#^	(1.37–3.32)
	8–9		−	−	−	−
	9–10		0.61 ^#^	(0.52–0.73)	0.83	(0.48–1.44)
	≥ 10		0.84	(0.56–1.25)	1.67	(0.57–4.86)

Statistically significant (^$^ p < 0.05, * p < 0.01, ^#^ p < 0.001)

Table 3 shows the correlation between OSAS symptoms and daytime sleepiness. Loud snoring and breathing pauses but not snorts and gasps, were significantly correlated with both mild and particularly severe daytime sleepiness. Multivariate logistic regression analysis with loud snoring, snorts and gasps, or breathing pauses as independent factors revealed a correlation between mild or severe daytime sleepiness and loud snoring (mild: OR = 2.21, p < 0.001; severe: OR = 3.15, p < 0.001), snorts and gasps (mild: OR = 2.69, p < 0.001; severe: OR = 7.39, p < 0.001), and breathing pauses (mild: OR = 2.66, p < 0.001; severe: OR = 7.53, p < 0.001).

**Table 3:** OSAS-related symptoms associated with daytime sleepiness in childhood and adolescence.

			Mild sleepiness		Severe sleepiness	
			Odds ratio	(95% CI)	Odds ratio	(95% CI)
Sex	Male		−	−	−	−
	Female		1.23 ^#^	(1.11–1.37)	1.03	(0.78–1.36)
Grade level	Elementary school	1	−	−	−	−
		2	0.78	(0.60–1.01)	0.56	(0.22–1.40)
		3	0.90	(0.70–1.15)	1.11	(0.51–2.39)
		4	1.01	(0.79–1.29)	0.75	(0.32–1.74)
		5	0.98	(0.76–1.25)	1.54	(0.75–3.15)
		6	1.05	(0.81–1.35)	1.45	(0.69–3.05)
	Junior high school	7	1.45 *	(1.13–1.87)	2.18 ^$^	(1.07–4.45)
		8	1.48 *	(1.15–1.91)	2.27 ^$^	(1.12–4.62)
		9	1.46 ^#^	(1.11–1.92)	2.22 ^$^	(1.07–4.59)
Snoring	Absent		−	−	−	−
	Present		2.07 ^#^	(1.77–2.43)	2.48 ^#^	(1.70–3.62)
Snorts and gasps	Absent		−	−	−	−
	Present		1.35	(0.85–2.16)	2.06	(0.87–4.87)
breathing pauses	Absent		−	−	−	−
	Present		1.59 ^$^	(1.05–2.40)	3.19 *	(1.48–6.91)
Total sleep time (h)	< 6		2.22 ^#^	(1.68–2.92)	11.49 ^#^	(6.95–18.97)
	6–7		2.54 ^#^	(2.07–3.10)	5.05 ^#^	(3.09–8.28)
	7–8		1.43 ^#^	(1.23–1.68)	2.11 ^#^	(1.36–3.29)
	8–9		−	−	−	−
	9–10		0.61 *	(0.51–0.72)	0.81	(0.47–1.41)
	≥ 10		0.83	(0.56–1.24)	1.65	(0.57–4.82)

* Statistically significant (^$^ p < 0.05, * p < 0.01, ^#^ p < 0.001).

## Discussion

This large-scale survey evaluated sleepiness, TST, and their association with prevalence of OSAS among school-age children (6–15 years) in Japan. The results revealed that independent of short sleep duration, presence of p-OSAS—defined based on the presence of loud snoring, snorts and gasps, or breathing pauses—was significantly correlated with daytime sleepiness in children. The correlation was particularly high between severe p-OSAS and severe daytime sleepiness.

Previous surveys targeting parents have reported a prevalence of OSAS or other sleep-related breathing disorders in children in the range of 4% to 11% [20–22]. Consistent with these studies, the present survey study found a prevalence of p-OSAS of 9.5% in elementary and middle-school age children.

Obesity is said to be a major cause of OSAS in adults, whereas lymphoid hyperplasia and adenoid and tonsillar hypertrophy are thought to be the major causes of childhood OSAS [37]. The incidence of OSAS is particularly high in children aged 2–8 years, which is around the time when the prevalence of lymphoid hyperplasia and adenoid and tonsillar hypertrophy peaks. However, a recent study has reported that besides anatomical problems in the upper respiratory tract such as adenoid and tonsillar hypertrophy, obesity and inflammation also contribute to childhood OSAS [38]. Also consistent with previous studies, OSAS peaked among children aged 6–10 years (grades 1–5) in this study, and the rate gradually decreased thereafter. Furthermore, while one study reported no sex-related difference in the prevalence of OSAS in children [39], other studies including ours found higher rates in boys than girls [20, 40].

The prevalence of childhood OSAS has been as low as 1% to 5.8%, however, in studies using objective diagnostic measures such as polysomnography and pulse oximetry [18–20]. This suggests that some children with p-OSAS in our study might have had much milder disorders such as upper airway resistance syndrome instead. Nevertheless, the clinical significance of our study is that we were able to identify children with possible OSAS using simple clinical characteristics such as loud snoring, which are easy for parents to recognize and would thus provide a valuable opportunity for children to undergo thorough examination and treatment for daytime sleepiness.

In the case of OSAS in adulthood, sleep is frequently interrupted by WASO, reducing the duration of rapid eye movement (REM) sleep and slow wave sleep in non-REM sleep, thereby causing daytime sleepiness. Unlike OSAS in adults, OSAS in children is rarely associated with arousal accompanied by increased brain wave activities, and therefore WASO occurs less frequently, maintaining sleep architecture [41, 42]. Moreover, while excessive daytime sleepiness is the chief complaint of OSAS in adults, it occurs less frequently in children and thus emphasizes the contribution of other behavioral factors such as impaired attention, hyperactivity, and aggressiveness [30, 33] [43]. For example, in a study of 508 healthy school-age children, excessive daytime sleepiness was associated with obesity, asthma, anxiety/depression, and difficulty falling asleep, but not OSAS [43]. However, the findings of other studies suggest an association between excessive daytime sleepiness and moderate-to-severe OSAS in school-age children [30] [44]; for example, despite no significant difference in daytime sleepiness between control subjects and all 108 pediatric patients with OSAS (mean age, 7 years), daytime sleepiness was observed in the severe OSAS group with an apnea-hypopnea index of ≥ 10 (times/h) [30]. The merit of the above four studies is that they examined children with a definitive diagnosis of OSAS based on polysomnography. However, because of the limited sample sizes and inconsistent findings among the studies, it remains controversial whether daytime sleepiness among school-age children is largely attributable to OSAS.

A limitation of the present study is that survey data were used to make a presumptive diagnosis of OSAS among children. However, because this large-scale study obtained data from ≥ 25000 children, it is reasonable to think that p-OSAS is an independent risk factor for daytime sleepiness in children. Previous studies have indicated that sleep debt also contributes to daytime sleepiness in pediatric patients with OSAS. Recently, sleep duration of adults in the United State has been reported to increase and this seems to be the "first signs of success in the fight against sleep deficiency" [45]. However, sleep duration among U.S. adolescents is decreasing in association with new media screen time [46]. The shortening of sleep duration among children and adolescents in recent years is an important ongoing problem. Indeed, short TST was strongly correlated with daytime sleepiness in the present study. In particular, there was an extremely strong correlation between severe daytime sleepiness and TST < 7 h. Even after adjusting for sleep duration, p-OSAS was significantly correlated with daytime sleepiness, especially severe daytime sleepiness.

From a clinical standpoint, the effect of OSAS on cognitive function in children should not be overlooked. Diffusion tensor images from children with OSAS have revealed low mean diffusivity in the dentate gyrus, the part of the hippocampus essential for neurogenesis and cognition, and lower mean diffusivity was associated with lower verbal learning [47]. Furthermore, childhood OSAS is reported to be associated with behavioral dysregulation (e.g., aggressiveness, impulsivity, and hyperactivity) [24], [48] and cognitive dysfunction (particularly attention, executive function, motor function, learning, and memory) [49]. An association has also been reported between cognitive function and the apnea-hypopnea index, an indicator of severity of OSAS in children with OSA [50] and improved academic achievement in children with OSAS after adeno-tonsillectomy [51]. This association suggests that worsening daytime sleepiness contributes, at least in part, to poor psychomotor performance in children with OSAS. It is therefore important for family members and school teachers to pay attention to OSAS-related symptoms such as loud snoring and daytime sleepiness in school-age children to ensure early detection and treatment of OSAS.

In this study, of the 3 OSAS-associated symptoms, loud snoring and breathing pauses were extracted as independent factors associated with daytime sleepiness. The association was lost for snorts and gasps after adjustment with all three symptoms as independent variables. This suggests that snorts and gasps are not highly specific to OSAS or are associated with the other two symptoms. For parents, loud snoring may be the most important and easiest OSAS indicator to look out for. According to a review article on SAS in children aged 6–13 years (a similar age group as in our study), 17% of children snored approximately 1 time per a week, 7.0% to 10.9% snored more frequently, and 2% snored all the time [20]. Similarly, 8.7% of children aged 6–15 years snored loudly ≥ 2 times per week in our study. Furthermore, snoring occurred less frequently with increasing age in the children in our study, consistent with the findings of a previous study [52].

The clinical practice guideline for the Diagnosis and Management of Childhood Obstructive Sleep Apnea Syndrome published by the American Academy of Pediatrics in 2012 [53] recommends that all children and adolescents should be screened for snoring. This suggests that snoring is the most important diagnostic indicator of OSAS. Primary snoring (PS, snoring without apnea or hypopnea) has been considered harmless because it is not associated with nocturnal awakening or changes in blood oxygen saturation levels or body mass index [54]. However, given findings that PS can cause neurocognitive dysfunction in children [55], more attention has been directed to snoring as a symptom too important to overlook.

## Conclusions

This large-scale epidemiological survey investigated duration of sleep and the contribution of OSAS to daytime sleepiness in elementary and middle school-age children in Japan. OSAS was found to be an independent factor for the appearance of daytime sleepiness in this age group. Among OSAS-associated symptoms, loud snoring and breathing pauses may be useful clinical markers for children with severe daytime sleepiness. Children with severe daytime sleepiness may be screened effectively by using the questionnaire utilized in this study [35] or the questionnaire that screens for sleep deficiency and the prevalence of OSAS among pre-school and elementary school children [56] [57].
